# OGRDB: a reference database of inferred immune receptor genes

**DOI:** 10.1093/nar/gkz822

**Published:** 2019-09-30

**Authors:** William Lees, Christian E Busse, Martin Corcoran, Mats Ohlin, Cathrine Scheepers, Frederick A Matsen, Gur Yaari, Corey T Watson, The AIRR Community, Andrew Collins, Adrian J Shepherd

**Affiliations:** 1 Institute of Structural and Molecular Biology, Birkbeck College, University of London, London WC1E 7HX, UK; 2 Division of B Cell Immunology, German Cancer Research Center, 69120 Heidelberg, Germany; 3 Department of Microbiology, Tumor and Cell Biology, Karolinska Institute, Box 280, 171 77 Stockholm, Sweden; 4 Department of Immunotechnology, Lund University, Medicon Village, S-223 81 Lund, Sweden; 5 Center for HIV and STIs, National Institute for Communicable Diseases of the National Health Laboratory Service, Sandringam, Gauteng 2131, South Africa; 6 Antibody Immunity Research Unit, School of Pathology, University of the Witwatersrand, Johannesburg 2050, South Africa; 7 Public Health Sciences, Fred Hutchinson Cancer Research Center, Seattle, WA 98109-1024, USA; 8 Faculty of Engineering, Bar Ilan University, Ramat Gan 5290002, Israel; 9 Department of Biochemistry and Molecular Genetics, University of Louisville School of Medicine, Louisville, KY 40202, USA; 10 School of Biotechnology and Biomolecular Sciences, University of New South Wales, Sydney, New South Wales 2052, Australia

## Abstract

High-throughput sequencing of the adaptive immune receptor repertoire (AIRR-seq) is providing unprecedented insights into the immune response to disease and into the development of immune disorders. The accurate interpretation of AIRR-seq data depends on the existence of comprehensive germline gene reference sets. Current sets are known to be incomplete and unrepresentative of the degree of polymorphism and diversity in human and animal populations. A key issue is the complexity of the genomic regions in which they lie, which, because of the presence of multiple repeats, insertions and deletions, have not proved tractable with short-read whole genome sequencing. Recently, tools and methods for inferring such gene sequences from AIRR-seq datasets have become available, and a community approach has been developed for the expert review and publication of such inferences. Here, we present OGRDB, the Open Germline Receptor Database (https://ogrdb.airr-community.org), a public resource for the submission, review and publication of previously unknown receptor germline sequences together with supporting evidence.

## INTRODUCTION

The genes of B-cell and T-cell antigen receptors (IG, TR) lie in some of the most structurally complex and polymorphic regions of vertebrate genomes. Because of their repetitive nature, the presence of many copy number variants, and the variation between individuals, the IG and TR genomic loci are problematic to study via standard high-throughput genomic approaches. For example, short-read surveys of human genetic variation such as the 1000 Genomes Project (1) remain challenging to interpret in these loci, to the extent that it is unclear whether such approaches can reliably deliver information on IG and TR germline variation (2, see also https://www.internationalgenome.org/faq/why-only-85-genome-assayable). An important consequence is that there are gaps in the current reference sets of IG germline genes and alleles—important gaps in human reference sets, and profound gaps in the sets of all other species, including those of medical and agricultural importance. Many of the sequences underlying the human germline set curated by IMGT (the international ImMunoGeneTics information system (3)), for example, were derived in the 1980s and 1990s from a small number of samples, primarily from either Caucasians or individuals of unknown ethnicity. The full extent of variation among human populations is not well understood and may be substantially underestimated (4–7). In contrast to studies of the human leukocyte antigen (HLA) (8) and the killer-cell immunoglobulin-like receptor (KIR) genes (9), there is little understanding of the common haplotypes of receptor genes. Similar, and possibly deeper, issues are arising in other species. As examples, extensive variation in IG heavy chain (IGH) genes has recently been reported between inbred laboratory mouse strains (10,11), while fish species important for food production exhibit substantial and complex genome and IG region-specific gene duplication (12).

Knowledge of IG gene variation is important. Polymorphism in the human IGHV1-69 gene has been shown to affect the antibody response to influenza A, with implications for vaccine design (13). Similar stereotyped immune responses have been observed in other infectious diseases and in contexts such as cancer and allergy (14–17). The analysis of the high-throughput sequencing of adaptive immune receptor repertoires (AIRR-seq) depends on an accurate germline set in order to identify clonal lineages and to correctly understand the impact of specific germline deletions and polymorphisms on the immune response. Gaps and erroneous sequences in reference sets therefore have a potentially detrimental impact on the development of effective diagnostic and therapeutic strategies (18).

In recent years, methods have been published through which personalized germline repertoires (identifying the set of germline receptor alleles expressed in the repertoire of a specific subject) can be inferred from AIRR-seq datasets (19–23). The personalized germline repertoire (referred to hereafter as a *genotype*) of any given person may be composed of previously unknown alleles as well as those already present in reference sets. Its inference from next-generation sequencing (NGS) provides a means through which high-throughput techniques can be applied to the problems of novel allele identification and population-level genetics. The AIRR Community (www.airr-community.org) - a network of over 300 practitioners in the field of AIRR-seq - and the IG, TR and MH Nomenclature Sub-Committee (IMGT-NC) (http://www.imgt.org/IMGTindex/IUIS-NC.php) of the International Union of Immunological Societies (IUIS)—recently reached agreement on a process whereby inferred genes and alleles would first be reviewed by Inferred Allele Review Committees (IARCs) under the auspices of the AIRR Community, and then submitted to IMGT-NC for their consideration (24). The first alleles were submitted for review in late 2018, and the first nine human IGHV genes were affirmed by the human IARC and accepted into IMGT in May 2019. Approximately 50 more are before the review committee, pending final confirmation of supporting data, and formation of IARCs for non-human species is in progress.

Review of inferred alleles is made in the context of individual AIRR-seq based genotypes, together with the accession numbers and details of underlying International Nucleotide Sequence Database Collaboration (INSDC) depositions. Ensuring data quality, tracking the progress of reviews and presenting the outcome to the community transparently was initially daunting, and it soon became apparent that computational support would be necessary. OGRDB (the Open Germline Receptor Database: https://ogrdb.airr-community.org) was developed to meet this need. It provides full supporting evidence for published alleles, recording the repertoires in which they have been observed and preserving references and history as additional information is received. Submitted alleles must be fully supported by records in public sequence repositories such as NIH Genbank and SRA, and links to these records are provided by OGRDB. For database users, OGRDB provides both the complete set of inferred alleles that have been affirmed through the IARC process, and also the inferred genotypes in which they were found. OGRDB may be viewed on large, medium or even small screen devices. Registration is freely available but only required for making new submissions: all published data is available without the need to register or log in.

## DATABASE METHODS AND RESULTS

### Inferred sequences

On the Sequences tab, OGRDB presents a browsable list of inferred alleles affirmed by the IARC review process, and, where a sequence has been accepted by IMGT, the canonical name allocated by IMGT. The Affirmation Level indicates the number of independent affirmations of the sequence by the IARC, up to a limit of three. The full set of sequences can be downloaded either in FASTA format, or in a provisional AIRR Community-defined format, which contains enriched metadata. Clicking on a sequence name provides a detailed view of the sequence record, including, importantly, the supporting evidence submitted to IARC that underlies the inferred sequence. Sequence entries are versioned: the Notes and History tabs provide details of IARC’s review, and any updates made to the record.

### Submissions and inferred genotypes

Supporting evidence for inferred sequences is contained in one or more submissions. Submissions underlying a particular sequence can be accessed by clicking on the Submission IDs in the Evidence section of the sequence record. Alternatively, the Submissions tab of OGRDB provides a browsable view of all published submissions.

**Figure 1.**
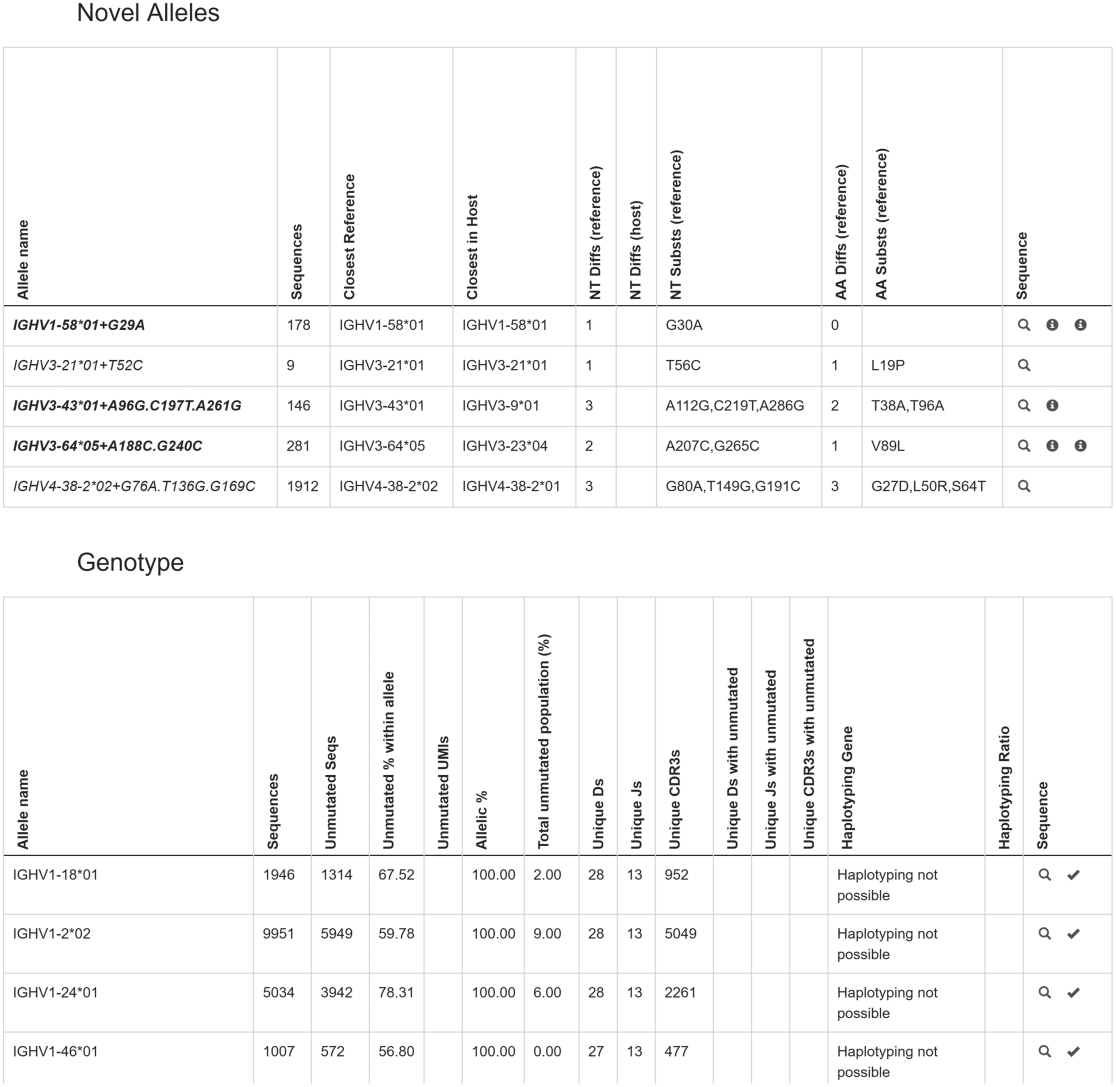
Partial screenshot of a genotype panel showing the statistics provided for inferred alleles, and, beneath, the statistics provided for all alleles (see Table 1 for a description of the information contained in these tables).

**Figure 2.**
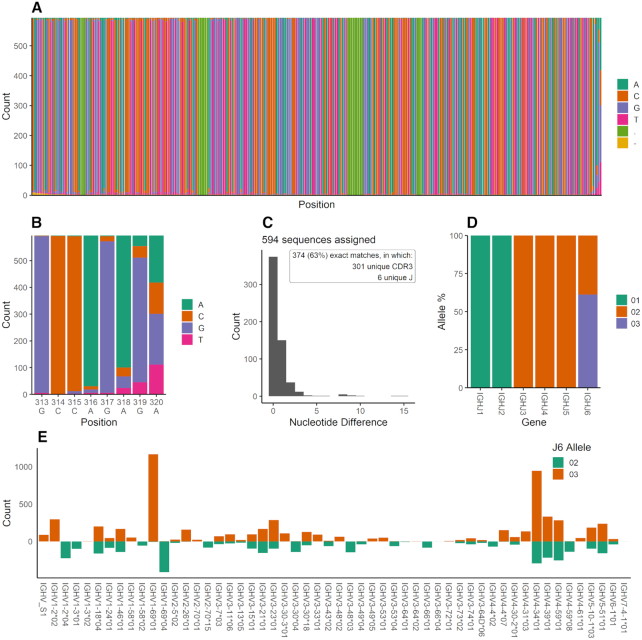
Illustrative plots produced by OGRDB analysis scripts from supported inference tools. These can be provided as part of a novel allele submission, or used independently by researchers interested in exploring the usage characteristics of an AIRR-seq repertoire. (**A**) IMGT alignment of all sequences assigned to a novel allele (IMGT gaps shown as ‘.’, base calls with low confidence as ‘-'), showing, in this case, a high-quality underlying dataset. (**B**) for V-genes, a ‘zoom’ of the previous plot showing the final 8 nucleotides at the 3′ end and illustrating the effect of the recombination process on the difficulty associated with inference of the final nucleotide of the IGHV gene based on the underlying data (27) (for J-genes, a similar plot is shown of the 5′ end). (**C**) a histogram, similar to that produced by IgDiscover (21), showing the reads assigned to the allele, distributed by the number of nucleotide differences to the reference (or inferred reference) sequence. (**D**) For the analysis of novel V-genes, a plot showing the usage of J-alleles within each J-gene, which can be used to identify heterozygosity (28). In this case, the subject is heterozygous in the IGHJ6 gene. For the analysis of novel J-genes, a similar plot of V-gene allele usage is provided. (**E**) where heterozygosity may be present, a plot is provided showing the number of reads of each V-gene, split by their usage of the heterozygous allele (in this case alleles *02 and *03 of IGHJ6). The novel allele IGHV_S1 is found exclusively in association with the IGHJ6*03 and no other alleles of IGHV_S1 were identified in the genotype: the exclusive association with *03 therefore provides additional support for the novel inference.

A submission reports the analysis of one or more AIRR-seq repertoires. The analysis is provided in the form of an inferred genotype and accompanying information. The genotype lists the set of genes within a particular locus that lead to expressed productive sequences in the repertoire. For each gene, it provides usage statistics, including its observed frequency and statistics on its usage in combination with other genes (Figure 1). OGRDB supports the derivation of a standardized genotype format from a number of analytical tools: conversion scripts and guidance for submitters are available on the website. Alongside the genotype itself, the scripts provide a range of plots that can be used by the submitter and by reviewers to assess the strength and quality of the novel inferences (Figure 2): these can be attached to a submission for additional information.

The repertoires underlying a submission are published in an INSDC repository such as the NIH Sequence Read Archive (SRA). Records for novel sequences, and extracted reads from the repertoire(s) that specifically support them, are also deposited. For ease of use and to facilitate review, OGRDB retrieves and displays metadata from NIH repositories. The sequences of reference alleles in each genotype are checked against the current IMGT reference set, and any discrepancies are identified. Human alleles are also checked against IgPdb (http://cgi.cse.unsw.edu.au/∼ihmmune/IgPdb/information.php), a repository of suspected polymorphisms in human IG genes. Although IgPdb remains a useful resource for accessing past inferences, the database has been largely inactive in recent years. This resource also differs from OGRDB in that alleles submitted to IgPdb do not require accompanying data that support the inferences. Submissions are not subject to expert review/evaluation and curation, and users of the database are unable to access evidence in support of the inferences. As a consequence of the lack of curation, IgPdb almost certainly includes sequences that have been reported in error. OGRDB also represents a significant improvement over IgPdb because tracking and cross-referencing maintains database correctness over time. For example, a novel sequence that is subsequently accepted by IMGT will still be identified in the underlying genotype as a novel sequence with the name assigned to it by the submitter, but it will be tagged with a note to show that it now matches a sequence in the IMGT reference set.

**Table 1.** Information provided in the OGRDB standardized genotype

Field	Description
sequence_id	Identifier of the allele (either IMGT, or the name assigned by the submitter to an inferred gene)
sequences	Overall number of sequences assigned to this allele
closest_reference	For inferred alleles, the closest reference gene and allele, as inferred by the tool
closest_host	For inferred alleles, the closest reference gene and allele that is in the subject's inferred genotype
nt_diff	For inferred alleles, the number of nucleotides that differ between this sequence and the closest reference gene and allele
nt_diff_host	For inferred alleles, the number of nucleotides that differ between this sequence and the closest reference gene and allele that is in the subject's inferred genotype
nt_substitutions	For inferred alleles, comma-separated list of nucleotide substitutions (e.g. G112A) between the sequence and the closest reference gene and allele. IMGT numbering is used for V-genes, and number from start of coding sequence for D- or J- genes.
aa_diff	For inferred alleles, the number of amino acids that differ between this sequence and the closest reference gene and allele
aa_substitutions	For inferred alleles, the list of amino acid substitutions (e.g. A96N) between the sequence and the closest reference gene and allele. IMGT numbering is used for V-genes, and number from start of coding sequence for D- or J- genes.
unmutated_sequences	The number of sequences exactly matching this unmutated sequence
assigned_unmutated_frequency	The number of sequences exactly matching this allele divided by the number of sequences assigned to this allele, *100
unmutated_umis	The number of molecules (identified by Unique Molecular Identifiers) exactly matching this unmutated sequence (if UMIs were used)
allelic_percentage	The number of sequences exactly matching the sequence of this allele divided by the number of sequences exactly matching any allele of this specific gene, *100
unmutated_frequency	The number of sequences exactly matching this sequence divided by the number of sequences exactly matching any allele of any gene, *100
unique_vs	The number of V allele calls (i.e. unique allelic sequences) found associated with this allele
unique_ds	The number of D allele calls (i.e. unique allelic sequences) found associated with this allele
unique_js	The number of J allele calls (i.e. unique allelic sequences) found associated with this allele
unique_cdr3s	The number of unique CDR3s found associated with this allele
unique_vs_unmutated	The number of V allele calls (i.e. unique allelic sequences) associated with unmutated sequences of this allele
unique_ds_unmutated	The number of D allele calls (i.e. unique allelic sequences) associated with unmutated sequences of this allele
unique_js_unmutated	The number of J allele calls (i.e. unique allelic sequences) associated with unmutated sequences of this allele
unique_cdr3s_unmutated	The number of unique CDR3s associated with unmutated sequences of this allele
haplotyping_gene	The gene or genes from which haplotyping was inferred, where haplotyping is possible (e.g. IGHJ6)

Provision of statistics for each allele in the personalized genotype (both reference alleles and novel alleles) allows the novel inferences to be considered in the context of overall gene usage (usage frequency, exact unmutated matches, association with distinct CDR3 and so on), and also provides useful aggregate information on overall gene usage.

### Submission and review process

This report focuses on the available published data; however, we encourage researchers who have suitable data sets to submit inferred alleles for review. Submission is online via OGRDB, and full details are provided on the site. OGRDB provides a submission and review workflow that supports multiple IARCs covering different species and loci, tracking the progress of all submissions and providing versioning and tracking of published sequences. The software is open source and may be of interest to other groups requiring a system for submission, review and publication.

## DISCUSSION

Our vision for OGRDB is to provide a rich and accessible record of observed receptor gene sequences, including not only the sequences themselves, but also detailed attribution and underlying information on their prevalence. We also aim to support and enhance the productivity of expert review, given the likely explosion of new information as high-throughput methods become increasingly available. While coverage today is limited to sequences inferred from AIRR-seq records, OGRDB contains the necessary fields to support genomic information, including annotation of non-coding regions, and we are interested to explore its extension to records derived from long-read sequencing, which can offer a partial (although to date not a complete) solution to the assembly problems inherent in high-volume genomic sequencing of the receptor loci.

The novel allele submission process is necessarily rigorous, requiring supporting information to be deposited in databases of record. Sequence sets supporting V-gene inferences must be full-length, excluding many data sets available today from consideration. We recognize the success and long-term impact of OGRDB depends greatly on the willingness of AIRR-seq data generators to submit allele inference data and supporting information. We will explore avenues to reduce the submission burden through the use of automated pipelines, and to leverage non-compliant data sets to provide additional support for previously affirmed inferences. VDJbase (https://www.vdjbase.org) (reported separately in this issue) is a database of inferences derived via a computational pipeline from a wider set of repertoires. We will develop integration between the two, and will explore opportunities to identify those datasets analysed within VDJbase which contain good candidate submissions for OGRDB and IARC review. By using results from the VDJbase pipeline, we can encourage submission of candidate sequences and reduce the burden on submitters. We intend to develop the two resources alongside one another to provide a rich and useful resource to the community.

Finally, while we have focussed on IG genes in this description and in the work of the current review committees, at least one analytical tool is available for TR gene inference (20), and OGRDB has been developed to accommodate the review and publication of both. We are keen to extend the scope of review to additional species and loci, and would welcome approaches from any groups interested in participating.

## CONCLUSION

The study of the adaptive immune system is of high clinical importance. While it has been facilitated by the widespread adoption of high-throughput techniques such as AIRR-seq (25,26), existing genomic resources have been hampered by the complexity of the regions concerned, and do not represent the diversity of human and animal populations. The development of high-quality, publicly available reference sets is a key aim of the AIRR Community. The presentation of inferred alleles through OGRDB is an important part of that vision. The partnerships now formed between OGRDB, IMGT, and VDJbase will further ensure the success of this initiative and increase its long-term impact.

As the application of AIRR-seq becomes even more pervasive in both the research and clinical arenas, we expect that the use of germline databases will also evolve. For example, it is likely that the storage of variant information beyond the coding regions will also offer important value to the community. With the initial foundation of OGRDB now established, we anticipate that OGRDB will be able to take on some of these extended initiatives. In addition, it will be important to begin understanding the prevalence of allelic variants stored in OGRDB and IMGT at the scale of populations and species. The concerted development of OGRDB with VDJbase, which is currently focused on providing rich information on AIRR-seq genotypes and alleles in human populations, will facilitate critical cross-talk between these two databases and ultimately provide a deeper view of IG and TR genetic diversity, and the importance of this diversity to the immune response.

## DATA AVAILABILITY

No registration or sign-on is required to access published data on OGRDB. All published information is freely available under a Creative Commons CC0 License. The source code is published at https://github.com/airr-community/ogre and is available under a European Union Public License v1.2.

## Supplementary Material

gkz822_Supplemental_File
