# Supplementary material for: OGRDB: a reference database of inferred immune receptor genes
Source: Nucleic Acids Res. 2019 Sep 30;48(D1):D964–70. doi: 10.1093/nar/gkz822 (PMC6943078; doi:10.1093/nar/gkz822)
Supplement: gkz822_Supplemental_File [file gkz822_supplemental_file.pdf]

OGRDB – a reference database of inferred immune receptor genes

List of AIRR Community members endorsing this manuscript

Adam Buntzman, University of Arizona
Anne Eugster, CRRD TU Dresden
Bjoern Peters, La Jolla Institute for Immunology
Brian Corrie, Simon Fraser University
Brian G. Pierce, University of Maryland Institute for Bioscience and Biotechnology Research
Camila H. Coelho, National Institute of Allergy and Infectious Diseases, Institutes of Health, Bethesda, MD, USA
Chaim Schramm, Vaccine Research Center, National Institute of Allergy and Infectious Diseases, NIH, Bethesda, MD, USA
Christopher Tipton, Emory University
Collin Joyce, The Scripps Research Institute
Daniel Douek, National Institutes of Health
David G. Coffey, Fred Hutchinson Cancer Research Center
Davide Bagnara, University of Genoa, Department of Experimental Medicine, Genoa, Italy
Duncan Ralph, Fred Hutchinson Cancer Research Center
Eline T. Luning Prak, Perelman School of Medicine, University of Pennsylvania
Eric Waltari, Chan Zuckerberg Biohub
Felix Breden, Simon Fraser University
George Blanck, University of South Florida
Gregory C. Ippolito, University of Texas at Austin
Henk-Jan van den Ham, ENPICOM B.V.
Inaki Sanz, Emory University
Inimary Toby, University of Dallas, Department of Biology
Jacob S. Sherkow, Harvard University
Jason Wilson, CDC
Jean-Philippe Buerckert, BISC Global
Johannes Trück, University Children's Hospital Zurich and the Children's Research Center, University of Zurich, Zurich, Switzerland
Lindsay Cowell,
Ludvig M. Sollid, University of Oslo
Manu Shankar-Hari, King's College London
Marcelo Macedo Brigido, Universidade de Brasilia
Ning Jiang, The University of Texas at Austin
Pei-Lung Chen, Graduate Institute of Medical Genomics and Proteomics, National Taiwan University
Ramit Mehr, Bar-Ilan University
Sandra C A Nielsen, Stanford University
Scott Christley, UT Southwestern Medical Center
Sol Efroni, Bar Ilan University
Steven Kleinstein, Yale School of Medicine
Susana Magadan, University of Vigo
Susanna Marquez, Yale University
Theam Soon Lim, Institute for Research in Molecular Medicine, Universiti Sains Malaysia
Thomas MacCarthy, Stony Brook University

Tilman Schneider-Hohendorf, University of Muenster, Germany, Department of Neurology with Institute of Translational Neurology
Uri Laserson, Icahn School of Medicine at Mount Sinai
Yoshinobu Koguchi, Earle A. Chiles Research Institute, Providence Cancer Institute
